# Bilateral hipoglossal nerve palsy in necrotizing otitis externa

**DOI:** 10.1016/S1808-8694(15)30115-4

**Published:** 2015-10-19

**Authors:** Adriano Santana Fonseca, Nilvano Alves de Andrade, Miguel Leal Andrade Neto, Vyrna Medeiros de Moura Santos

**Affiliations:** a Specialist in Otorhinolaryngology and Head and Neck Surgery - HC/UNICAMP; Assistant Professor of Head and Neck Surgery - Santa Casa de Salvador - Santa Izabel Hospital. Head of the Otorhinolaryngology Clinics of the Naval Hospital in Salvador. Dysphagologist and Head and Neck Surgeon - NOEV/Hospital - Bahia.; b M.S. in Surgery -UFBA and PhD in Otorhinolaryngology - USP; Otorhinolaryngology and Head and Neck Surgery Residency Program Coordinator - Santa Izabel Hospital and Head of the Santa Izabel Hospital - Santa Casa de Misericórdia da Bahia.; c Resident Physician in Otorhinolaryngology - Santa Izabel Hospital - Santa Casa de Misericórdia da Bahia.; d Resident Physician in Otorhinolaryngology - Santa Izabel Hospital - Santa Casa de Misericórdia da Bahia.

**Keywords:** otitis externa, necrotizing otitis, nerve palsy

## INTRODUCTION

Necrotizing otitis externa is a potentially lethal infection that starts in the external auditory canal and may progress to the skull base. It happens in elderly diabetic patients and is associated to a high morbi-mortality rate.^1^ The major causal agent is Pseudomonas aeruginosa, in 96 to 98% of the cases^2^.

The infection extends from the osteocartilaginous junction to the temporal bone by means of the Santorini fissures. The infection may progress towards the skull base and affect the facial, glossopharyngeal, vagus and accessory nerves. It may occasionally affect the hypoglossal, abducens and trigeminal nerves.^3^

Symptoms such as otalgia, headache, hypoacusis, otorrhea, in diabetic or immunosupressed patients are very relevant. Laboratory investigation reveals high ESR, with normal or mildly high white cell count. Clinical signs include ulceration on the floor of the EAC. CT scan shows bone destruction, and MRI shows both the location and extension of the infection, intracranial invasion and cranial nerve involvement. Scintigraphy with technetium and gallium has been used in order to assess cure criteria. Technetium scintigraphy is useful to diagnose osteitis, which is positive in cases of acute or chronic osteomyelitis, or trauma. It bears low specificity and may remain positive for one year^2^. Galium-67 scintigraphy is used in the follow up and check of the therapeutic response, since galium has great affinity for acute phase leucocytes and proteins.^2^

Germ culture is necessary for proper treatment, however one should not wait for its result in order to start treatment. Two empirical antibiotics are used: aminoglycosides together with ciprofloxacin of cephtazidime. Alternative drugs are cefepime, cefoperazone, imipenem and aztreonam. Treatment lasts between 4 and 6 weeks.^1^ Otalgia reduction or cessation is an important control parameter. Most patients may be clinically treated, and surgery bears controversies, such as: progressive pain, cranial neuropathy and granulation persistence in the EAC. Mastoidectomy may be carried out, however in some cases it does not prevent skull base disease extension.^3^

## CASE REPORT

AAB, 71 years old, male, from Salvador, hypertensive and diabetic, came to us in August 20, 2004, complaining of two months of right side otalgia. He had used antibiotics (cephalexin, moxifloxacin e gatifloxacin) without improvement, and started having very sharp pain. The exam showed signs of otitis media, with EAC thickening, without ulceration. CT scan (July 2004) showed right side mastoid and middle ear opacification. Audiometry showed mixed hypoacusis.

Thinking of mastoiditis, the patient was treated with ciprofloxacin for 21 days and evolved with symptoms remission.

In October of 2004, otalgia returned and a new CT scan showed a rupture in the mastoid’s external cortical bone.

The patient was submitted to mastoidectomy and the anatomical study revealed chronic unspecific inflammation and positive culture for Pseudomonas. The patient was started on ciprofloxacin 1500 mg/daily for 6 weeks.

Diabetes compensation, pain control and epithelization of the canal wall down cavity was the treatment result. That is why we did not order scintigraphy for initial control.

**Figure 1:**
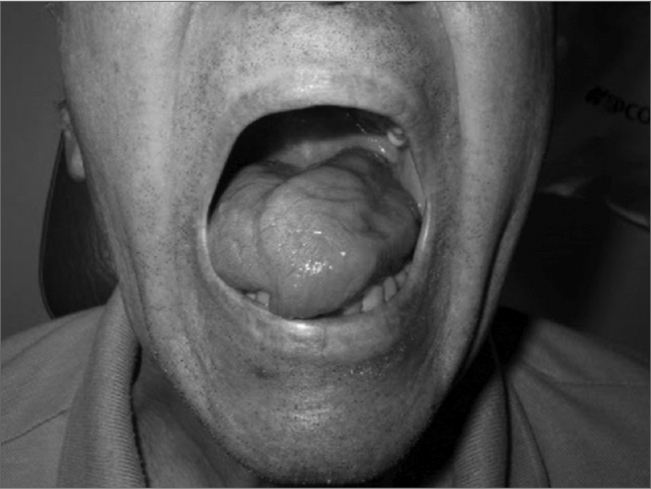
Tongue protrusion attempt with an infection in the retropharyngeal area.

He returned after 4 months complaining of headache and tongue paralysis (Figure 1). He was admitted and a new CT scan revealed contrast uptake from the petrous apex to the retropharynx, and opacification of the canal wall down cavity. Technetium scintigraphy was non-conclusive.

Right side mastoid cavity biopsy and culture, together with that from the retropharynx through nasal endoscopy revealed unspecific inflammatory process in a pseudomonas culture.

The patient remained in the hospital with cefepime and amycacin for 8 weeks. He was discharged symptomless, with normal CT scan and controlled glucose levels.

10 weeks afterwards, he came back with headaches, worsening in his dysphagia and contralateral hypoacusis and otorrhea. A new CT scan showed bilateral mastoiditis and retropharynx mass extending to the oropharynx.

Blood culture and left ear secretion culture revealed Pseudomonas and a biopsy of the oropharynx muscles showed unspecific chronic inflammatory process. The patient remained in the hospital for one week and was discharged with a central line for outpatient use of cefepime 4g/daily for 6 months. The patient was treated and is under follow up for 10 months now, with signs of tongue reinnervation.

## DISCUSSION

Necrotizing otitis externa, for the first time described by Chandler, in 1968, is an aggressive infection of the external ear, involving the skin, cartilage and temporal bone.

Intravenous antibiotics is the treatment of choice, as long as there is no osteomyelitis - in these cases surgery is indicated.^1^, ^2^, ^3^

It is very rare to see NOE involving the neck muscles and the contralateral mastoid.^3^ A benign outcome after skull base osteomyelitis is also statistically very rare, since the mortality rate in these cases is of 60-80%.^3^
